# Leadership transition

**DOI:** 10.1002/btm2.10422

**Published:** 2022-10-21

**Authors:** Samir Mitragotri

**Affiliations:** ^1^ John A Paulson School of Engineering & Applied Sciences Harvard University Allston Massachusetts USA

*Bioengineering & Translational Medicine* is completing its seventh year of publication in 2022. The journal was launched with the mission of providing a platform for featuring cutting‐edge research and technologies at the interface of engineering and medicine, with a particular focus on translation. Many transformative medical technologies find their roots in engineering, and the journal aims to provide a forum to celebrate the role of technological innovation in medicine.

The first issue of *Bioengineering & Translational Medicine* was published in March 2016 which included seven articles focused on nanomedicine. The journal quickly established itself in the expansive landscape of bioengineering journals. Over the last 5 years, the journal has witnessed tremendous growth; the number of submitted manuscripts has grown by more than 10‐fold and the manuscript download rate has increased by 5‐fold. *Bioengineering & Translational Medicine* articles have been downloaded in more than 200 countries, thus attesting to the global reach of the journal. The journal received its first impact factor in 2020 and the impact factor now stands at 10.68. These metrics speak to the solid foundation that the journal has built in its nucleation phase.

As the journal enters its next growth phase, I have decided to step down as the Editor in Chief. It has been a pleasure and privilege to serve as the founding Editor in Chief of *Bioengineering & Translational Medicine*. I am deeply thankful to our advisory board who provided strong support and guidance in the journal's formative days. I am especially grateful to our Associate Editors, Profs. Elizabeth Nance, Kaushal Rege, Zongmin Zhao, Hadley Sikes, and Amir Sheikhi who have been instrumental in operating the journal during its rapid growth on multiple fronts, especially in handling the ever‐increasing manuscript submission volume. Sincere thanks also go to Dr. Aaron Anselmo who served as an Associate Editor in the early days of the journal. I express my sincere appreciation for the AIChE and Wiley teams for their strong support to *Bioengineering & Translational Medicine*. I also express my deep gratitude to our authors who trusted the journal in its formative days and submitted their manuscripts. Without their trust, the journal would not have gotten off to such a strong start.

*Bioengineering & Translational Medicine* reflects the needs and views of the broad translational bioengineering community. As such, a leadership transition will provide additional perspectives for the journal's future growth. Now is a natural time for a transition in leadership. I am thankful to AIChE for appointing me as the Editor in Chief to launch *Bioengineering & Translational Medicine* and after a successful launch, it is my pleasure to hand off this responsibility to the next Editor in Chief to take the journal to new heights. I am delighted that Prof. Elizabeth Nance has agreed to serve as the interim Editor in Chief of the journal. Prof. Nance has already made tremendous contributions to the journal as Associate Editor and has launched multiple initiatives of high importance to the journal. I am looking forward to her leadership as the interim Editor in Chief.

To the readers, authors, and reviewers of the journal, I would like to reiterate my deep appreciation for your staunch support to *Bioengineering & Translational Medicine* over the past 7 years, and I hope that you continue to support the journal in years ahead.

## AUTHOR CONTRIBUTIONS

**Samir Mitragotri:** Conceptualization (lead); writing – original draft (lead); writing – review and editing (lead).

### PEER REVIEW

The peer review history for this article is available at https://publons.com/publon/10.1002/btm2.10422.

